# Transcriptome analysis in roots and leaves of wheat seedlings in response to low-phosphorus stress

**DOI:** 10.1038/s41598-019-56451-6

**Published:** 2019-12-24

**Authors:** Jun Wang, Qin Qin, Jianjun Pan, Lijuan Sun, Yafei Sun, Yong Xue, Ke Song

**Affiliations:** 1 Eco-environmental Protection Research Institute, Shanghai Academy of Agricultural Sciences, Shanghai, 201403 China; 2 Shanghai Scientific Observation and Experimental Station for Agricultural Environment and Land Conservation, Shanghai, 201403 China; 3 Shanghai Environmental Protection Monitoring Station of Agriculture, Shanghai, 201403 China; 4 Shanghai Engineering Research Centre of Low-carbon Agriculture (SERLA), Shanghai, 201403 China; 5 Shanghai Key Laboratory of Protected Horticultural Technology, Shanghai, 201403 China; 6 College of Resources and Environmental Sciences, Nanjing Agricultural University, Nanjing, 210095 China

**Keywords:** Transcriptomics, Abiotic

## Abstract

Low phosphorus availability is a major abiotic factor constraining wheat growth. The molecular mechanisms of the wheat whole genome under low-phosphorus stress are still unclear. To obtain information on gene expression in wheat seedlings under low-phosphorus stress, transcriptome sequencing was performed on roots and leaves. The results showed that 2,318 (1,646 upregulated and 672 downregulated) transcripts were differentially expressed in the leaves, and 2,018 (1,310 upregulated and 708 downregulated) were differentially expressed in the roots. Further analysis showed that these differentially expressed genes (DEGs) were mainly enriched in carbon fixation in photosynthetic organs and in carbon metabolism, photosynthesis, glyoxylate and dicarboxylate metabolism and plant-pathogen interaction in both leaves and roots. These pathways were mainly associated with environmental adaptation, energy metabolism and carbohydrate metabolism, suggesting that the metabolic processes were strengthened in wheat seedlings under low-phosphorus stress and that more energy and substances were produced to resist or adapt to this unfavourable environment. This research might provide potential directions and valuable resources to further study wheat under low-phosphorus stress.

## Introduction

Phosphorus (P) is an essential macronutrient for plant growth and development and plays a key role in the regulation of energy metabolism and the synthesis of nucleic acids and membranes^1,2^. Though abundant in soil, it is often limited for plants due to its low bioavailability^3^. Low-phosphorus stress seriously restricts crop growth and reduces the yield of crops^4,5^. To obtain high yields, excessive quantities of P-fertilizer are applied by farmers. Unfortunately, crops can only utilize up to 30% of inorganic phosphate (Pi) from fertilizers because the inaccessible forms are formed from its reaction with iron-aluminium oxides and calcium carbonate compounds^6,7^. The remaining phosphorus is fixed in the soil or transferred to groundwater, lakes and oceans through farmland drainage and surface runoff, leading to water eutrophication and algal blooms^8,9^. Understanding the molecular mechanism of crops’ responses to low-phosphorus stress and improving their phosphorus use efficiency is an important means to solve these problems.

Transcriptome profiling using next-generation sequencing technologies can detect the molecular mechanisms of plant responses to abiotic stress^10,11^. Some studies have been conducted on plant responses to low-phosphorus stress. In barley (*Hordeum vulgare* L.), transcriptome analysis revealed that many genes were significantly upregulated or downregulated in response to low-phosphorus stress, and the DEGs were mainly involved in phosphorus metabolism, such as phospholipid degradation, sucrose synthesis, phosphorylation/dephosphorylation, hydrolysis of phosphoric enzymes and post-transcriptional regulation^12^. It was also reported that the DEGs were enriched in the oxidation-reduction process, carbohydrate metabolic process, biosynthetic process, and tricarboxylic acid cycle of oat roots under low-P treatment^13^. To better understand these processes, the DEGs were also investigated under low-phosphorus stress in other crops through transcriptome analysis, such as maize^14^, rice^15^, and soybean^16^. Transcriptome analysis has greatly improved insights into the sophisticated molecular mechanisms regulating phosphorus homeostasis in numerous cultivated crops. However, little research has been conducted on the response of wheat to low-phosphorus stress.

Wheat is one of the main cultivated crops in the worldwide food system. To meet the needs of the world’s growing population, the grain yield of wheat must increase at an average annual rate of approximately 2% in a limited area of cultivated land^17^. However, wheat yield is frequently threatened by low-phosphorus stress, especially in acidic and alkaline soils in tropical and subtropical regions^18,19^. Therefore, improving the phosphorus use efficiency and biomass yield of wheat under low-phosphorus conditions has great practical significance.

In this paper, we analysed the wheat responses to low-phosphorus stress using transcriptome analysis. The transcriptome profile will provide more information on the wheat gene sequence related to phosphorus efficiency, and the identification of DEGs following low-phosphorus stress can deepen our understanding of the genetic variation of wheat under low-phosphorus stress and suggest strategies to enhance their phosphorus use efficiency and biomass production with less fertilizer application.

## Results

### Plant growth

Low-phosphorus stress significantly inhibited the growth of wheat seedlings. Under low-phosphorus stress, plant height, shoot dry weight, root dry weight and total root length of wheat seedlings were significantly lower than those of the CK, by 24.76%, 26.75%, 45.66% and 45.10%, respectively (Table 1). Additionally, the total phosphorus uptake of low-phosphorus-treated seedlings was significantly decreased by 69.28% compared with the CK (Table 1).

**Table 1:** Effects of low-phosphorus stress on growth parameters of wheat seedlings.

Treatments	Plant height (cm)	Shoot dry weigh (mg/plant)	Root dry weigh (mg/plant)	Total root length (cm/plant)	Total phosphorus uptake (mg/plant)
CK	26.58 ± 0.60a	30.77 ± 0.27a	23.17 ± 0.57a	57.83 ± 1.31a	0.153 ± 0.009a
-P	20.00 ± 0.68b	22.54 ± 0.36b	12.59 ± 0.66b	31.75 ± 0.82b	0.047 ± 0.007b

CK: control; -P: low-phosphorus stress. The data are from the average of 15 seedlings; for each parameter, the mean values (±standard error) are presented.

Note: Different letters (a, b) indicate that there are significant differences at the 0.05 level according to Tuckey’s test.

### Transcriptome sequencing datasets

To understand the molecular mechanism of the response of wheat seedlings to low-phosphorus stress, the gene expression of wheat seedlings under low-phosphorus stress was investigated by transcriptome sequencing. In total, 43.22, 47.08, 45.63 and 41.25 million raw reads were obtained by Illumina sequencing of the -P-L, -P-R, CK-L and CK-R cDNA libraries, respectively (Table 2). After filtering low-quality reads, 42.80 (-P-L), 46.46 (-P-R), 45.10 (CK-L), and 40.73 million clean reads (CK-R) (Q20 > 95.07%) were generated as shown in Table 2, the GC content of which was between 54.26% and 55.38%. The error rate of all clean data per sample was controlled below 0.02%. A total of 71.06~84.43% of these clean reads were mapped to the wheat genome (Table 2). Of the mapped data, 91.94~93.69% of the clean reads were uniquely mapped to the genome.

**Table 2:** Quality of sequencing. CK-L: control leaf; CK-R: control root; -P-L: low-phosphorus-stressed leaf; -P-R: low-phosphorus-stressed root.

Sample	Raw Read No.	Clean Reads No.	Clean Reads %	Q20%	GC %
CK-L	45633102	45096752	98.82	95.24	55.38
CK-R	41250032	40726872	98.73	95.24	54.62
-P-L	43218210	42795728	99.02	95.67	55.13
-P-R	47080182	46460684	98.68	95.07	54.26

### Functional annotation of unigenes

**Figure 1:**
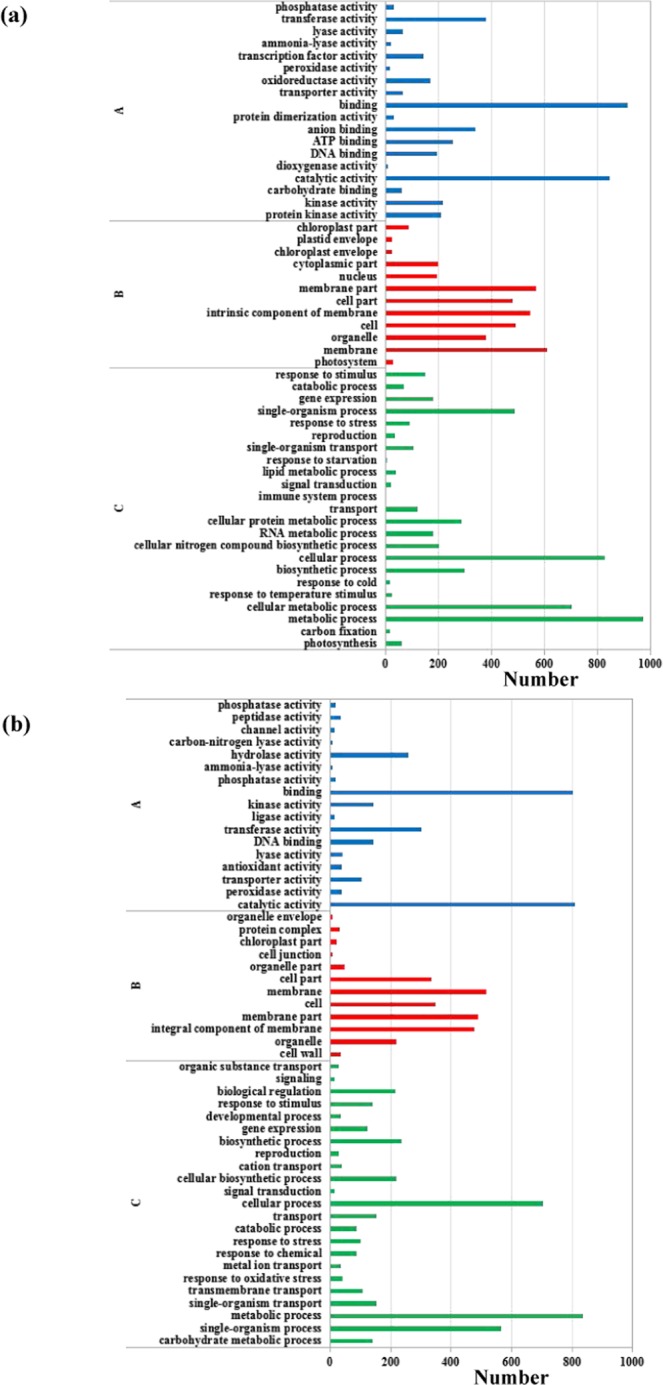
GO classification of assembled unigenes in wheat leaves (**a**) and roots (**b**). (A) Molecular function. (B) Cellular component. (C) Biological process.

The sequences of unigenes were searched against the Gene Ontology (GO) database and the Kyoto Encyclopedia of Genes and Genomes (KEGG) database. GO analysis showed that genes were defined in three major functional categories: molecular function, cellular component and biological process. In total, these unigenes were further classified into 53 and 52 subcategories in the leaves and roots, respectively. Unigenes involved in binding, catalytic activity, membrane, membrane part, intrinsic component of membrane, metabolic process, cellular process and single-organism process in leaves and roots were the most enriched subcategories (Fig. 1a,b).

**Figure 2:**
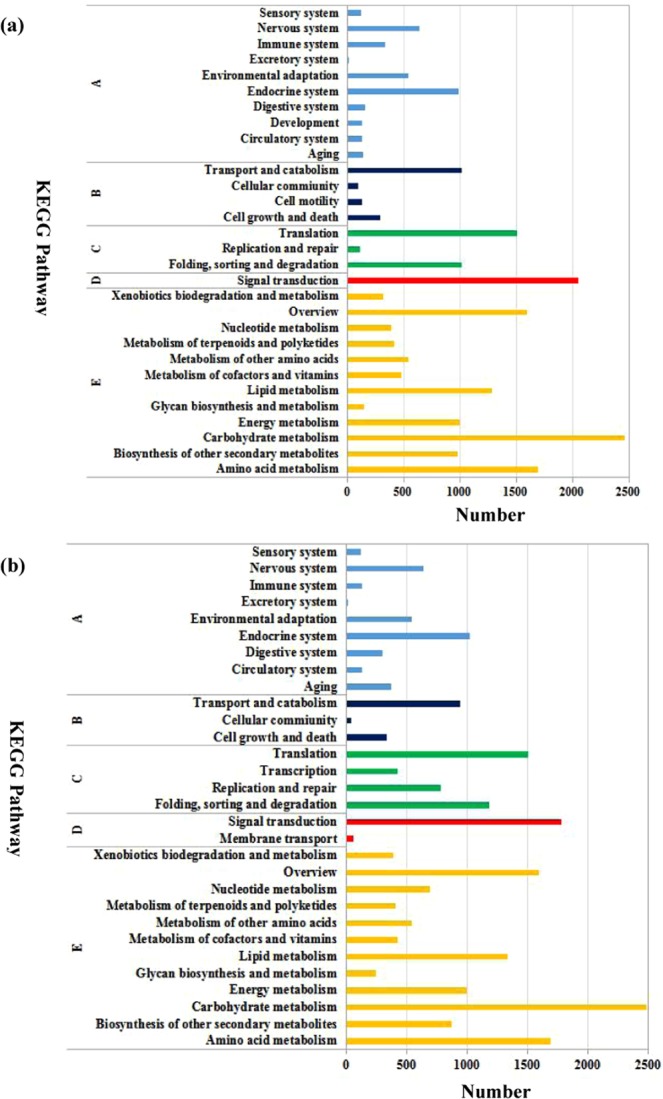
KEGG classification of assembled unigenes in wheat leaves (**a**) and roots (**b**). (A) Organismal systems. (B) Cellular processes. (C) Genetic information processing. (D) Environmental information processing. (E) Metabolism.

The KEGG database was used to identify the metabolic pathways that may be fully or partially represented by annotated coding sequences of unigenes. A total of 18414 unigenes in leaves (9330 unigenes) and roots (9084 unigenes) were assigned to five categories containing 30 KEGG pathways. Among them, carbohydrate metabolism, signal transduction, amino acid metabolism, overview and translation in leaves and roots were the five most strongly represented pathways (Fig. 2a,b).

### Identification and analysis of differentially expressed genes

**Figure 3:**
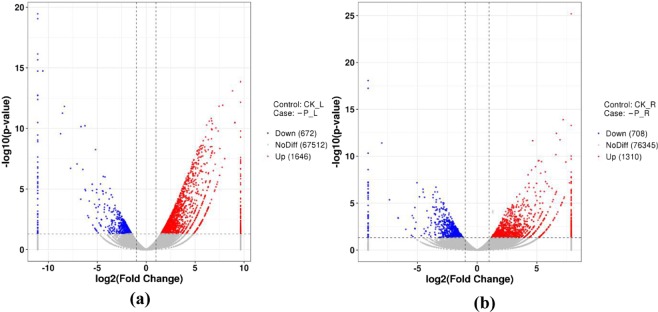
Volcano plots of DEGs between the control and low-phosphorus-stressed wheat seedling leaves (**a**) and roots (**b**). The two vertical dotted lines are twice the difference threshold, and the horizontal dotted line represents a p-value of 0.05. The red dots indicate the upregulated genes in this group, the blue dots indicate the downregulated genes in this group, and the grey dots indicate the non-significantly differentially expressed genes.

DESeq software was used to discriminate genes that were differentially expressed in response to low-phosphorus stress. An absolute value of log 2FoldChange > 1 and *p*-value < 0.05 were set as the threshold to judge the significance of gene expression differences between low-phosphorus and sufficient-phosphorus conditions. A total of 4336 differentially expressed genes (DEGs) were identified in wheat seedlings under low-phosphorus stress. Among the DEGs, 1,646 genes were upregulated and 672 genes were downregulated in low-phosphorus leaf samples (Fig. 3a), while 1,310 genes were upregulated and 708 genes were downregulated in low-phosphorus root (Fig. 3b).

### GO enrichment analysis of DEGs

**Figure 4:**
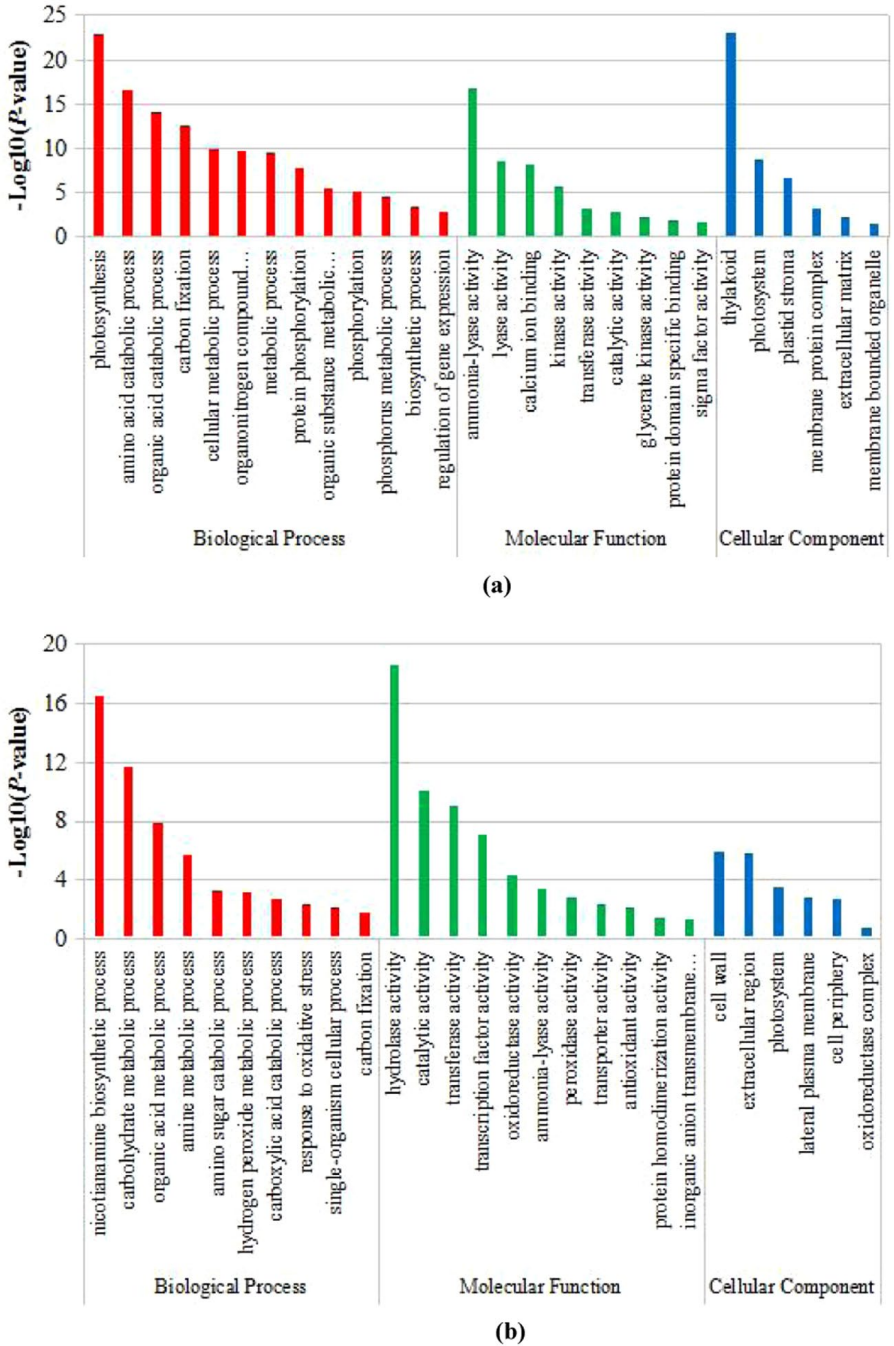
Gene Ontology (GO) enrichment analysis of differentially expressed genes (DEGs) in wheat leaves (**a**) and roots (**b**) under low-phosphorus stress.

To understand the functions of the DEGs, GO enrichment analysis was performed in leaves and roots of wheat seedlings under low-phosphorus stress. All the DEGs were classified into three main functional categories containing 157 and 116 GO terms in leaves and roots, respectively. In leaves, the number of GO terms in biological process, cellular component and molecular function were 95, 30 and 32, respectively. Under biological process, the DEGs were significantly enriched in the terms photosynthesis, amino acid catabolic process and organic acid catabolic process. Under cellular component, the DEGs were significantly enriched in the terms thylakoid, photosystem and plastid stroma. Under molecular function, the DEGs were significantly enriched in the terms ammonia-lyase activity, lyase activity and calcium ion binding (Fig. 4a).

Among the root DEGs, the number of GO terms in biological process, cellular component and molecular function were 77, 7 and 32, respectively. Under biological process, the DEGs were significantly enriched in the terms nicotianamine biosynthetic process, carbohydrate metabolic process and organic acid metabolic process;. Under cellular component, the DEGs were significantly enriched in the terms cell wall and extracellular region. Under molecular function, the DEGs were significantly enriched in the terms hydrolase activity, catalytic activity and transferase activity (Fig. 4b).

### KEGG enrichment analysis of DEGs

**Figure 5:**
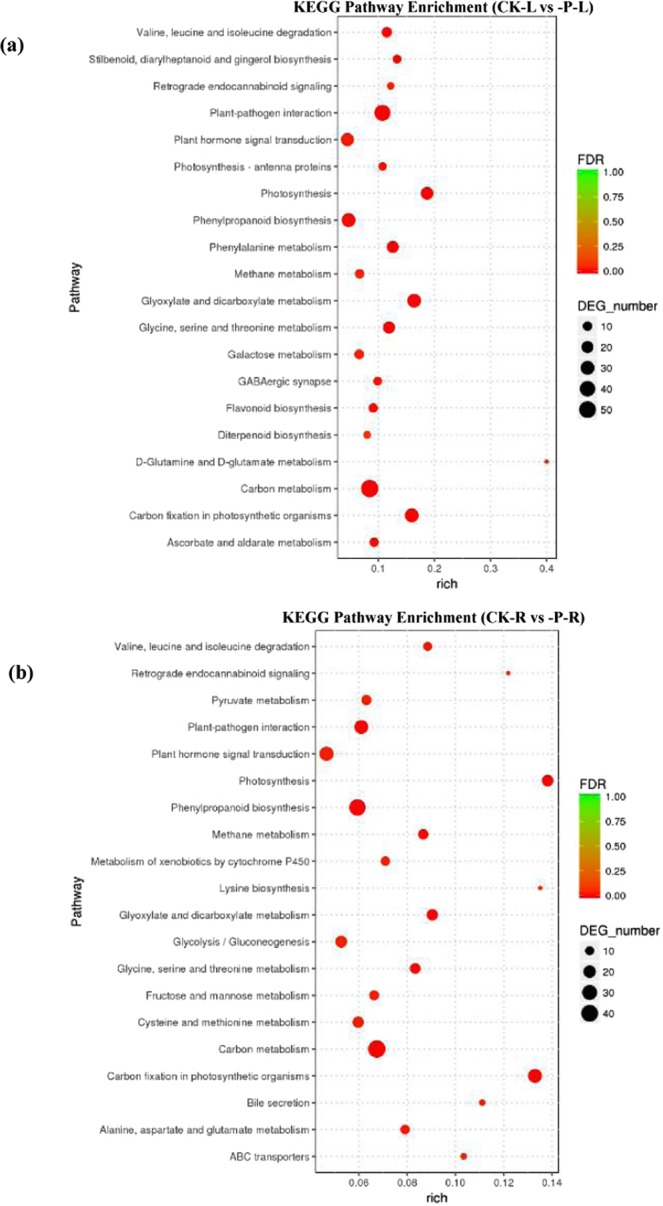
KEGG pathway enrichment analysis of differentially expressed genes (DEGs) between -P and CK treatments in wheat leaves (**a**) and roots. (**b**) The left Y-axis represents the top 20 pathways. The X-axis represents the percentage of DEGs belonging to the corresponding pathway. The sizes of bubbles represent the number of DEGs in the corresponding pathway, and the colours of the bubbles represent the enrichment p-value of the corresponding pathway.

To perform functional classification and analyse the pathway enrichment of DEGs in wheat seedlings under low-phosphorus stress, KEGG pathway enrichment analysis was carried out. In leaves, 848 DEGs were successfully assigned to 158 KEGG pathways. Among them, 37 pathways were significantly enriched (*p*-value < 0.05) in pathways categorized into three branches, namely, environmental information processing, metabolism and organismal systems, of which the DEGs enriched in metabolism were dominant. The 20 pathways with the most DEGs involved in the response to low-phosphorus stress in wheat seedlings are shown in Fig. 5. Plant-pathogen interaction, carbon fixation in photosynthetic organs, glyoxylate and dicarboxylate metabolism, carbon metabolism and photosynthesis were the most represented pathways in leaves (Fig. 5a).

In roots, a total of 803 DEGs were successfully assigned to 164 pathways. Cellular processes, environmental information processing, metabolism and organismal systems were the most represented pathways in roots. These functions (except for cellular process) were also among the most enriched in leaves (Fig. 5b).

### Validation of the DEGs by qRT-PCR

**Figure 6:**
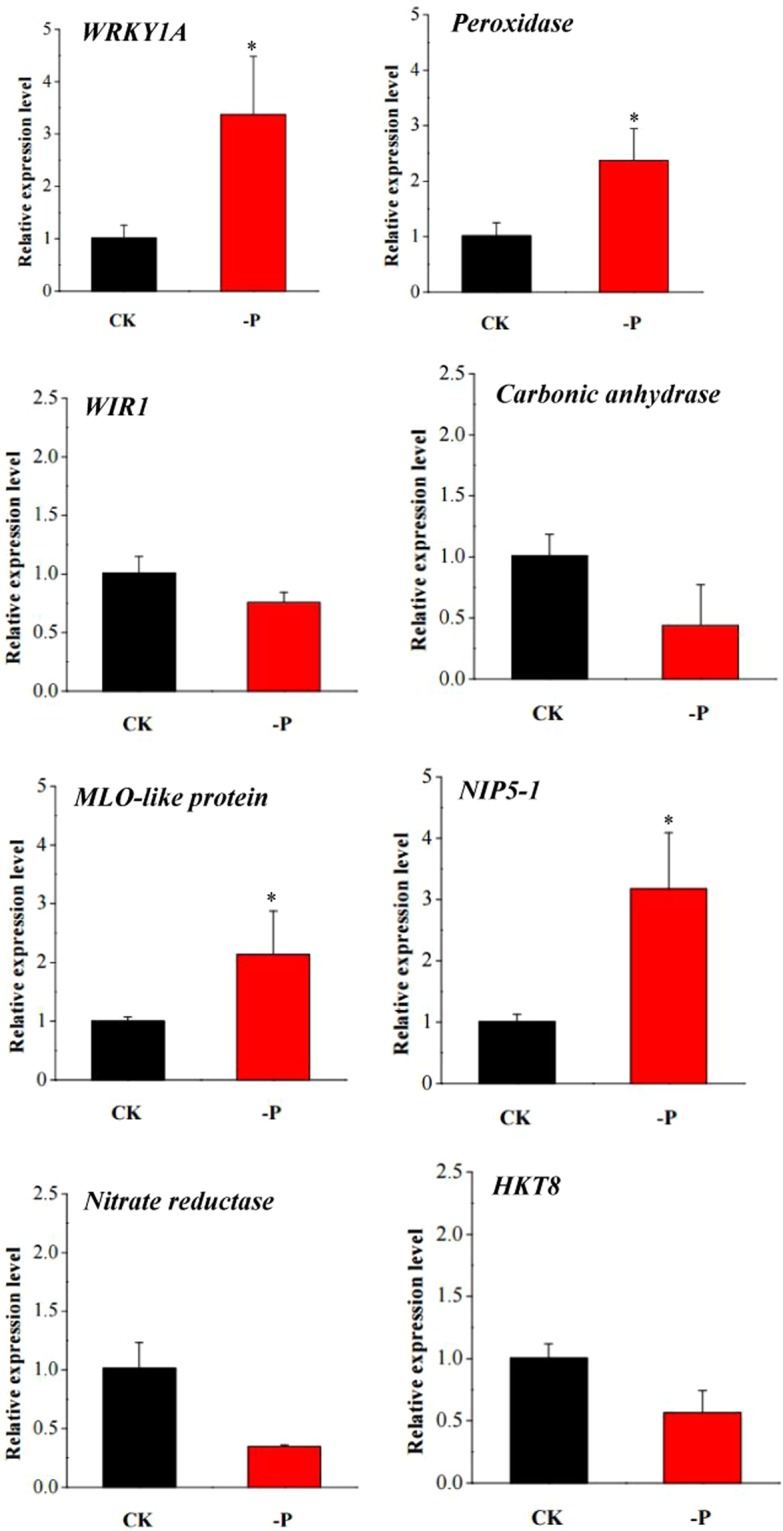
The relative gene expression of 8 randomly selected genes examined by qRT-PCR analysis. CK: control; -P: low-phosphorus stress. Data represent the mean ± SE (n = 3). Asterisks indicate significant differences compared to the control (t test, p-value < 0.05).

To further validate the reliability of the identified DEGs in response to low-phosphorus stress in wheat seedlings, eight genes, including four upregulated and four downregulated genes, were randomly selected from roots and leaves, and their expression in response to low-phosphorus stress was detected by quantitative reverse transcription-PCR (qRT-PCR) analysis. The results showed that there was good consistency between the expression levels of the eight genes analysed by qRT-PCR and their levels detected using RNA-seq (Fig. 6). Consequently, the qRT-PCR analysis results confirmed that the data we obtained from RNA-seq were trustworthy.

## Discussion

### Response to low-phosphorus stress by plant growth and phosphorus adsorption

A low-phosphorus or phosphorus-deficient environment has a significant impact on the morphology of plants^20,21^. Reymond *et al*. pointed out that the primary root length was reduced when *Arabidopsis thaliana* was grown under low-phosphorus conditions^22^. In addition, the shoot dry mass of *Arabidopsis thaliana* grown in a low-phosphorus environment was significantly reduced^23^. The shoot and root dry matter of chickpea plants were reduced equally when plants were subjected to low phosphorus supply^24^. In this study, low-phosphorus stress significantly decreased wheat biomass, especially root biomass. The inhibition of root growth led to a decrease in phosphorus adsorption (by 69.28% in comparison to the control). These changes suggest that low-phosphorus stress has a serious impact on wheat seedling growth and phosphorus adsorption.

### The key roles of potential DEGs in low-phosphorus tolerance in wheat seedlings

Phosphorus stress usually triggers dramatic molecular responses in plants. To gain insight into the molecular response mechanisms involved in low-phosphorus stress in wheat seedlings, RNA-seq analysis was performed. A total of 4,336 DEGs were identified from the whole wheat seedling in response to low-phosphorus stress, including 2,018 DEGs (1,310 upregulated and 708 downregulated) from roots and 2,318 DEGs (1,646 upregulated and 672 downregulated) from leaves. These results suggested serious changes in gene expression in wheat seedlings in response to low-phosphorus stress.

GO enrichment analysis showed that the greatest proportion of DEGs were enriched in the category biological process in both leaves and roots of wheat seedlings. DEGs were involved in photosynthesis, amino acid metabolism, organic acid metabolism and carbohydrate metabolism and were expressed highly in wheat seedlings. Many researchers have suggested that differentially expressed transcripts in these processes might play essential roles in plant adaptation to low-phosphorus stress. For example, evidence has shown that a large number of photosynthesis-related genes are differentially expressed in rice under low-phosphorus stress^25^. Phosphorus deficiency resulted in significant differences in the expression of genes associated with amino acid and organic acid metabolism in oat^26^. In addition, many DEGs involved in the process of carbon and energy conversion were identified under low-phosphorus stress^27,28^. Enzymes are biocatalysts that control many processes of metabolism, nutrition and energy conversion. Many genes related to enzymes will be activated to resist or adapt to adverse environments during plant growth and development. For example, the peroxidase, phosphate dehydrogenase and malate dehydrogenase genes were upregulated in rice under low-phosphorus stress^25,29^. Our study showed that genes related to transferase, oxidoreductase, ammonia-lyase and peroxidase activity were upregulated under low-phosphorus stress, which may provide the necessary material and energy for wheat seedlings to resist or adapt to low-phosphorus stress.

### Some candidate genes for plant low-phosphorus stress tolerance breeding

KEGG analysis showed that the DEGs were mainly involved in plant-pathogen interactions, carbon fixation in photosynthetic organisms, glyoxylate and dicarboxylate metabolism, carbon metabolism and photosynthesis in wheat seedlings under low-phosphorus stress. These pathways were also mainly related to environmental adaptation, energy metabolism and carbohydrate metabolism, mostly associated with material circulation and energy transfer. The results showed that the transport of substances and energy might be intensified in wheat seedlings under low-phosphorus stress, and such an adaptation will be significant for wheat’s resistance or adaptation to a low-phosphorus environment. The following discussion highlights some important KEGG pathways that are likely involved in mediating the wheat seedling response against low-phosphorus stress.

### Photosynthesis

Photosynthesis is an essential part of the life cycle of green plants and provides the necessary energy source for plant metabolism^30,31^. Phosphorus deficiency inhibits photosynthetic activity in plants by changing the photosynthetic phosphorylation process, and inhibition of the photosynthetic phosphorylation process can lead to slow metabolism, thus affecting crop growth and development^32^. In addition, ATPase and NADPH are involved in plant photosynthesis and play an important role in energy metabolism and material composition. The phosphorus concentration will directly affect their activities^33,34^. In this paper, many photosynthesis-related genes were significantly upregulated, including fructose-1,6-bisphosphatase, ferredoxin (Fd), Fd-NADP reductase and ATP synthase, which are involved in the process of photophosphorylation and energy metabolism. The results indicated that some enzymes related to photosynthesis may play an active role in regulating the early response to low-phosphorus stress and triggering the resistance response of wheat seedlings. Surprisingly, in this study, photosynthesis-related genes were identified in the roots of wheat under low-phosphorus stress. Li *et al*. showed that there are many photosynthesis-related DEGs in rice roots under low-phosphorus conditions^25^. It has been reported that it may be related to saving energy^35^, but this response mechanism needs further study.

### Carbon metabolism

Low-phosphorus stress has a certain effect on the expression of genes involved in carbon metabolism in wheat seedlings, especially the expression of genes related to glycolysis, the tricarboxylic acid (TCA) cycle, and the phosphorylation pathway. 6-Phosphofructokinase 1 (PFK1), pyruvate kinase (PK) and glyceraldehyde 3-phosphate dehydrogenase (GAPD3) are key enzymes involved in glycolysis, and the pathways catalysed by PFK1 and PK are irreversible. A previous study showed that genes related to glycolysis, such as PK and GAPD3, were involved in resistance to low-nitrogen and -phosphorus stress^36,37^. We found that many genes involved in glycolysis were significantly upregulated after low-phosphorus stress in this paper, and some were downregulated in wheat leaves, including PFK1 and GAPD3, but the expression of the GAPD3 and PK genes were upregulated in wheat roots. This result suggests that glycolysis was enhanced in roots under low-phosphorus stress and that many energy-producing genes were activated to resist low-phosphorus stress in wheat roots. Some genes that are involved in the TCA cycle were upregulated in leaves and roots, such as malate dehydrogenase (MDH) and isocitrate dehydrogenase (IDH). MDH genes maintaining glutathione were reduced for the functioning of glutathione S-transferase (GST). GSTs may act as binding proteins that sequester flavonoids in the vacuole for protection against environmental stresses^38^. Therefore, we hypothesized that the upregulated expression of MDH genes may play an important role in protecting wheat from a low-phosphorus environment. Protein phosphorylation occurs in many kinds of amino acids, most of which are serine. Protein phosphorylation plays a major role in signal transduction, is involved in the regulation of nearly all processes within the cell, and enables cells to rapidly respond to environmental changes by controlling the functional properties of proteins in response to external stimuli^39^. In this paper, we found that many serine-related genes and phosphotransferase genes involved in phosphorylation were upregulated in wheat roots. This result suggests that a low-phosphorus environment induces many phosphorylation-related genes to respond to it and participate in the transmission of stress signals. These data provide a basis for helping future organisms resist or adapt to this environment.

### Glycine, serine and threonine metabolism

The enhancement of the metabolome could improve the anti-stress ability of the plants, and one analysis of differentially expressed genes showed that serine metabolism under abiotic environmental stimuli was significantly higher than that under a normal growth environment^40,41^. In addition, changes in the expression of amino acid metabolism genes were observed in sorghum roots, suggesting that these genes can activate the nitrogen uptake and accumulation mechanism under nitrogen deficiency^42^. Wang *et al*. showed that some amino acid metabolism genes, including those of glycine, serine and threonine metabolism, were upregulated in oat roots under phosphorus deficiency^26^. Our research showed that the expression levels of genes related to the glycine, serine and threonine metabolism pathways were significantly upregulated in both wheat leaves and roots in response to low-phosphorus stress, and these genes were mainly related to amino acid metabolism. The differential expression of these genes might play an important role in protecting wheat seedlings from low-phosphorus stress, and we hypothesized that the upregulation of glycine, serine and threonine metabolism genes may also promote phosphorus uptake and accumulation in wheat seedlings.

### Plant-pathogen interaction

Plants are frequently affected by pathogen infections, and stripe rust and scab are common diseases of wheat. Plant pathogens, including bacteria, fungi, oomycetes and viruses, can cause plant diseases; however, a lack of nutrients can also lead to plant diseases^43^. To effectively defend against such infections, the mode of innate immunity in molecular pathways has developed in plants^44^. A previous study showed that once plants are invaded by pathogens, they trigger some proteins to respond to them, and defensive mechanisms are marshalled^45^. In this paper, many genes belonging to the plant-pathogen interaction pathway were significantly upregulated in both wheat leaves and roots, and these genes were mainly related to calcium-binding protein, calcium-dependent protein kinase and calmodulin. This suggests that some diseases inflict wheat seedlings under low-phosphorus stress and that genes encoding calcium-binding proteins, calcium-dependent protein kinase and calmodulin were activated to enhance the plant-pathogen interaction.

In conclusion, we conducted a comparative analysis of mRNA expression in roots and leaves of wheat seedlings under low-phosphorus stress by a transcriptomic approach. A total of 2,318 DEGs (1,646 upregulated and 672 downregulated) were identified in leaves, and 2,018 DEGs (1,310 upregulated and 708 downregulated) were identified in roots. Further annotation and analysis indicated that DEGs were mainly enriched in five main pathways involved in the wheat seedling response to low-phosphorus stress, which was mainly associated with metabolism processes. This suggests that the metabolism processes were strengthened in wheat seedlings under low-phosphorus stress and that more energy and substances were produced to resist or adapt to this unfavourable environment. These results have expanded our knowledge about the molecular mechanisms active in wheat seedlings under low-phosphorus stress, which will provide potential directions and valuable resources for further research on the wheat response to this stress.

## Materials and Methods

### Plant materials and growth conditions

A representative wheat (*Triticum aestivum* L.) cultivar was our first choice. WM 52, the breeding unit, was from the seed company of Suzhou City, Anhui Province. It was bred by sexual hybridization with Zhengzhou 8329 as a female parent and Wanmai 19 as a male parent. The variety is semi-wintery, and its advantages are high yield, high quality, cold resistance, disease resistance, lodging resistance and drought resistance. WM 52 is a commonly selected wheat variety in Shanghai and is widely planted in other parts of China. Therefore, WM 52 has good representativeness and practical significance as a test material. Wheat seeds were surface-sterilized with 75% ethanol for 1 min and then rinsed 3~4 times with distilled water, soaked in distilled water and germinated in a growth chamber at 25 °C in the dark for 24 h. The uniformly germinated seeds were selected and grown on moistened germination paper in a growth chamber at a day/night temperature of 20/15 °C (12 h photoperiod) with relative humidity of 75% and 3000 lx of light intensity. When seedlings grew to approximately 5 cm high, the seedlings were transplanted into plastic pots filled with improved Hoagland solution^46^ and were grown afterwards in an incubator at 20/15 °C (day/night) under a 12 h photoperiod until they showed two fully expanded leaves. During the growth period, seedlings were sprayed with distilled water on time.

### Low-phosphorus stress experiments

Two-leaf-stage seedlings were transferred to different conditions: phosphorus sufficiency (KH_2_PO_4_ 0.5 mM, CK) and low-phosphorus stress (KH_2_PO_4_ 0.05 mM, -P). Except for the phosphorus concentration, the other components of the solution under low-phosphorus stress were identical to those of the control. There were 18 seedlings per treatment with three biological replicates. The growth solutions were refreshed every 3 days. After 14 days of treatment under low-phosphorus stress, root and leaf tissues from different treatments were collected and immediately frozen in liquid nitrogen and then stored at −80 °C until RNA extraction.

### Determination of plant biomass and total phosphorus content

All tissues (root and leaf) were de-enzymed at 110 °C for 10 min and dried at 75 °C until their weight remained constant, to calculate the dry weight. A colorimetric method was used for determination of total phosphorus content^47^. All dried tissues were crushed and then digested at 250 °C for 1 h. The developer was then added, and colorimetric determination was carried out after 30 minutes.

### RNA isolation, cDNA library construction, and Illumina sequencing

Total RNA was isolated for tripled biological replicates from the sampled roots or leaves using the Trizol reagent (Invitrogen, Carlsbad, CA, USA) according to the manufacturer’s instructions. The quantity, quality and integrity of the total RNA were examined using a NanoDrop 1000 spectrophotometer (NanoDrop Technology, USA) and an Agilent 2100 Bioanalyzer (Agilent Technologies, Santa Clara, CA, USA). Magnetic beads with Oligo (dT) were used to isolate mRNA from the total RNA. The isolated mRNA mixed with the fragmentation buffer was cut into short fragments, and the mRNA fragments were generated into first-strand cDNA using reverse transcriptase and random hexamer primers. After that, second-strand cDNA was subsequently synthesized using first-strand buffer, DNA polymerase I and RNase H. The cDNA fragments were purified and washed with EB buffer for terminal reparation and poly(A) addition. The short fragments were subsequently connected with adapters. The suitable fragments (200 bp) were PCR-amplified for cDNA library construction. The library was sequenced using the Illumina HiSeq^TM^ platform (Shanghai Personal Biotechnology, Shanghai, China).

### Read filtration and assessment of differential gene expression

To obtain high-quality reads, reads with adaptor sequences, low-quality reads (with a quality score lower than 10%) and ambiguous ‘N’ nucleotides (with a ratio of ‘N’ more than 5%) were removed by the Cutadapt software. After that, a reference genome index was established using Bowtie2 software^48^, and the remaining paired-end clean reads were used to map the reference genome (http://www.ensembl.org/index.html) by TopHat 2.

Read numbers mapped to every gene were counted using HTSeq software. The RPKM (reads per kilobase per million reads) value per gene was calculated according to the gene length and mapped read count. Genes with RPKM > 1 were deemed expressed in the current study. Differential expression between the low-phosphorus and control groups was then analysed using the DESeq R package (http://www.bioconductor.org/packages/release/bioc/html/DESeq.html). An absolute value of log 2FoldChange > 1 and *p*-value < 0.05 were used as the thresholds to judge the significance of gene expression differences.

### Annotation and functional enrichment analysis of differentially expressed genes (DEGs)

Gene annotations and functional enrichment analysis, including Gene Ontology (GO) and Kyoto Encyclopedia of Genes and Genomes (KEGG) biological pathways, were used to identify which DEGs from root and leaf tissues after low-phosphorus treatment were significantly enriched in GO terms or biological pathways. Gene annotations against the GO database (http://geneontology.org/) were performed using the Blast2Go program. GO terms and biological pathways against the KEGG database (http://www.genome.jp/kegg) with a *p*-value < 0.05 were deemed to be significantly enriched in DEG analysis.

### Validation of transcriptome sequencing

To confirm the reliability of the RNA-seq results, quantitative real-time PCR (qRT-PCR) was performed using the SYBR^®^ Premix EX Taq kit on a Rotor-Gene 3000 real-time PCR detection system (Qiagen). Eight differentially expressed genes induced by low-phosphorus stress were randomly selected from roots and leaves for experimental validation (Table S1). *RLI* (Ta2776) was used as an internal control. Specific primers for real-time PCR were designed by Primer Premier 5.0 software (Premier Biosoft International), as shown in Table S1. All amplification programmes were as follows: 95 °C for 5 min, followed by 40 cycles at 95 °C for 15 s and 60 °C for 30 s. Three quantitative assays were performed on each cDNA. The relative expression levels of genes were calculated using the formula 2^−△△Ct^ ^49^.

## Supplementary information

Supplementary Information
