# Supplementary material for: Transcriptome analysis in roots and leaves of wheat seedlings in response to low-phosphorus stress
Source: Sci Rep. 2019 Dec 24;9:19802. doi: 10.1038/s41598-019-56451-6 (PMC6930268; doi:10.1038/s41598-019-56451-6)
Supplement: Supplementary file 1 — Supplementary Information [file 41598_2019_56451_MOESM1_ESM.pdf]

Transcriptome analysis in roots and leaves of wheat seedlings in response to low-phosphorus stress

Jun Wang[1,6†], Qin Qin[1,2,3,4,5†], Jianjun Pan[6], Lijuan Sun[1,2,3,4,5], Yafei Sun[1,2,3,4,5], Yong Xue[1,2,3,4,5,6*] & Ke Song[1,2,3,4,5*]

Table S1. Primer information for qRT-PCR

Primer	Forward (5′→3′)	Reverse (5′→3′)
WRKY1A	GCATGGACTGCCTTTTCGAG	GATGCTCTGTTCGTCGTCCT
Peroxidase	GAAGATGGACGAGTGGAAGATG	CAGACCTCTCACCTAATCCTAACA
WIR1	AGCCTCGCTCGAATCATCAG	GGTTAGGGTCGAAAGCACCA
Carbonic anhydrase	CCGCAACATCGCCAACAT	GAGTGAGAGGAGAGCCTTGATT
MLO-like protein	CTTCCTCATACACCTCACACTCT	ACCTTGGCGATGCTCACT
NIP5-1	ATTTCCACTCCCCGTTCCTG	GTTGGATCTGGGTCGATGCT
Nitrate reductase	CACCTCCACGCCCTTCAT	ATCCACGCCGACTCCTTG
HKT8	GACGACAGTGACCAACAAGT	ACGGCGATGAAGATGGCC
RLI	TTGAGCAACTCATGGACCAG	GCTTTCCAAGGCACAAACAT
